# Perceptions of safety during everyday travel shaping older adults’ mobility in Bengaluru, India

**DOI:** 10.1186/s12889-024-19455-0

**Published:** 2024-07-19

**Authors:** Divya Sussana Patil, Ajay Bailey, Sobin George, Lena Ashok, Dick Ettema

**Affiliations:** 1 Transdisciplinary Centre for Qualitative Methods, Department of Data Science, Prasanna School of Public Health, Manipal Academy of Higher Education, Manipal, Udupi, Karnataka 576104 India; 2 Department of Human Geography and Spatial Planning, Faculty of Geosciences, Utrecht University, Utrecht, The Netherlands; 3 Centre for Study of Social Change and Development, Institute for Social and Economic Change, Bengaluru, India; 4 Department of Social and Health Innovation, Prasanna School of Public Health, Manipal Academy of Higher Education, Manipal, Karnataka 576104 India

**Keywords:** Older adults, Perceptions of safety, Public transportation, Non-motorized transport, Mobility

## Abstract

**Background:**

In the context of socially sustainable urban development, comfortable, safe, and accessible public transport is crucial to motivating people to travel more sustainably. Using the framework given by Masoumi and Fastenmeier (2016) to examine the concepts of safety and security, we explore how perceptions of safety about different transport modes shaped the mobility of older adults in Bengaluru, India.

**Methods:**

In-depth telephonic interviews were conducted with 60 adults, aged 50 years and over, residing in urban Bengaluru, using a semi-structured in-depth interview guide to explore the perceptions of safety in different transport modes. Observations were conducted prior to the COVID-19 pandemic. Applying thematic analysis, we present how the perceptions of safety during their everyday travel shaped their mobility.

**Results:**

According to our research, older adults’ perception of safety during their everyday travel is shaped by past negative experiences with accidents, pickpocketing, theft of mobile phones, and chain snatching. In addition, the Covid-19 pandemic exacerbated the already existing inequalities, further limiting older adults’ mobility to carry out regular activities such as buying groceries, socialising, making a hospital visit, or going to work due to the fear of getting infected.

**Conclusion:**

Our findings indicate that the use of public transport needs to be encouraged among older adults by enhancing necessary safety features following the age-friendly cities framework. Furthermore, it can help policymakers develop transport polices, which suit the mobility needs of older adults.

## Background

An individual’s well-being and quality of life are closely linked with their ease of physical mobility. With advancing age, older adults experience certain health limitations such as cognitive decline, osteoarthritis, visual impairments etc. that affect their mobility [1]. Hence it is necessary to ensure that their mobility needs are met. Transport safety is an important aspect of transportation, which influences the mobility and well-being of older people [2, 3]. Often perceptions on lack of safety during travel results in stress, fear, and loneliness that negatively impacts on mobility, independence, and accessibility to social life, healthcare, and work [4, 5]. For many older adults, their daily activities are largely limited to a few trips for buying groceries, socializing, visiting a hospital, or commuting to work [6]. Participating in these activities are important for them to lead a healthy life [7, 8]. Evidence indicates that independent mobility enables older adults to engage in social activities, thus promoting health [9–11]. Whereas the inability to participate in social life due to low quality urban transport infrastructure results in depressive symptoms and social isolation, leading to poor health outcomes [12–14]. There is also evidence that older adults prefer not to use their cars or two-wheelers to carry out these activities due to cognitive and functional decline [15–17]. Hence, most of their trips may include walking, using public transport, hiring an intermediate transport, or depending on family and non-family members for mobility [18]. For this paper, we considered the World Health Organisation (WHO) definitions of well-being as “Wellbeing exists in two dimensions, subjective and objective. It comprises an individual’s experience of their life as well as a comparison of life circumstances with social norms and values” [19], Quality of Life (QoL) as “an individual’s perception of their position in life in the context of the culture and value systems in which they live and in relation to their goals, expectations, standards and concerns” [20], and ‘health’ as “a state of complete physical, mental and social well-being and not merely the absence of disease or infirmity.” [21].

More often the mobility of older people is limited due to lower perceptions of safety during everyday travel [22]. Falls and injuries while boarding/alighting from public transport and inside the transport mode due to negligence of the driver, and while walking on uneven or narrow pavements can lead to debilitating health conditions for older adults from a safety perspective [1]. Moreover, crimes such as theft while on public transit or walking, road traffic accidents, overcrowded public transport, and traffic congestion can limit older people’s ability to get out of the house, thereby negatively impacting their well-being [1, 23, 24]. Another key concern for older people is crossing roads during peak traffic hours. Although main road networks in cities usually have markings and signals for pedestrians crossing the street, pedestrians find it difficult to walk at a faster pace to match the traffic, resulting in them not being able to cross the road before the signal changes [25, 26]. Further, the behaviour of other road users who do not follow traffic rules makes crossing the road challenging [27]. Therefore, it is necessary to improve the design and infrastructure of the transport system to make older adults feel safe during their commute for daily activities. Additionally, lockdowns and restrictions due to COVID-19 pandemic created a sense of fear for older adults to use public transport modes [28]. In the city of Bengaluru, various measures were taken to stop the spread of infection. These include advising older adults not to leave their houses, suspending public transport services, removing fare concessions for older adults, thereby restricting older adults from using bus services [29]. This affected older adults from lower income groups who could not afford private or para-transit modes (for e.g., autorickshaw, cab). The fear of contracting infections also limited their use of mass-transit modes [30]. In order to safely run the public transportation without excluding the services for older adults, it is important to develop context-specific plans depending on the severity of the pandemic and number of older adults in a specific location for e.g., re-designing the seating arrangements to maintain safe distance, social distancing at bus stops, contactless ticketing system, door-to-door pick up and drop off for older adults, dedicated bus timings for older adults to reduce risk of exposure etc. [31, 32].

Existing studies have focused on the role of neighbourhood environments and their influence on active ageing (India and Columbia) [33, 34], the effects of new urban designs on public health [35], and perceptions of safety while walking (Canada) [36]. Older adults’ perceived lack of safety in transportation with respect to built environment features, public transport, and public transport stations and its effects on their mobility have not been explored in the South Asian context. Hence, in the present study we address the following research questions: How do older adults perceive safety in the transportation system? How did these perceptions of safety shape their mobility and transport mode choice in the city?

### Perceived safety shaping the mobility of older adults

**Fig. 1:**
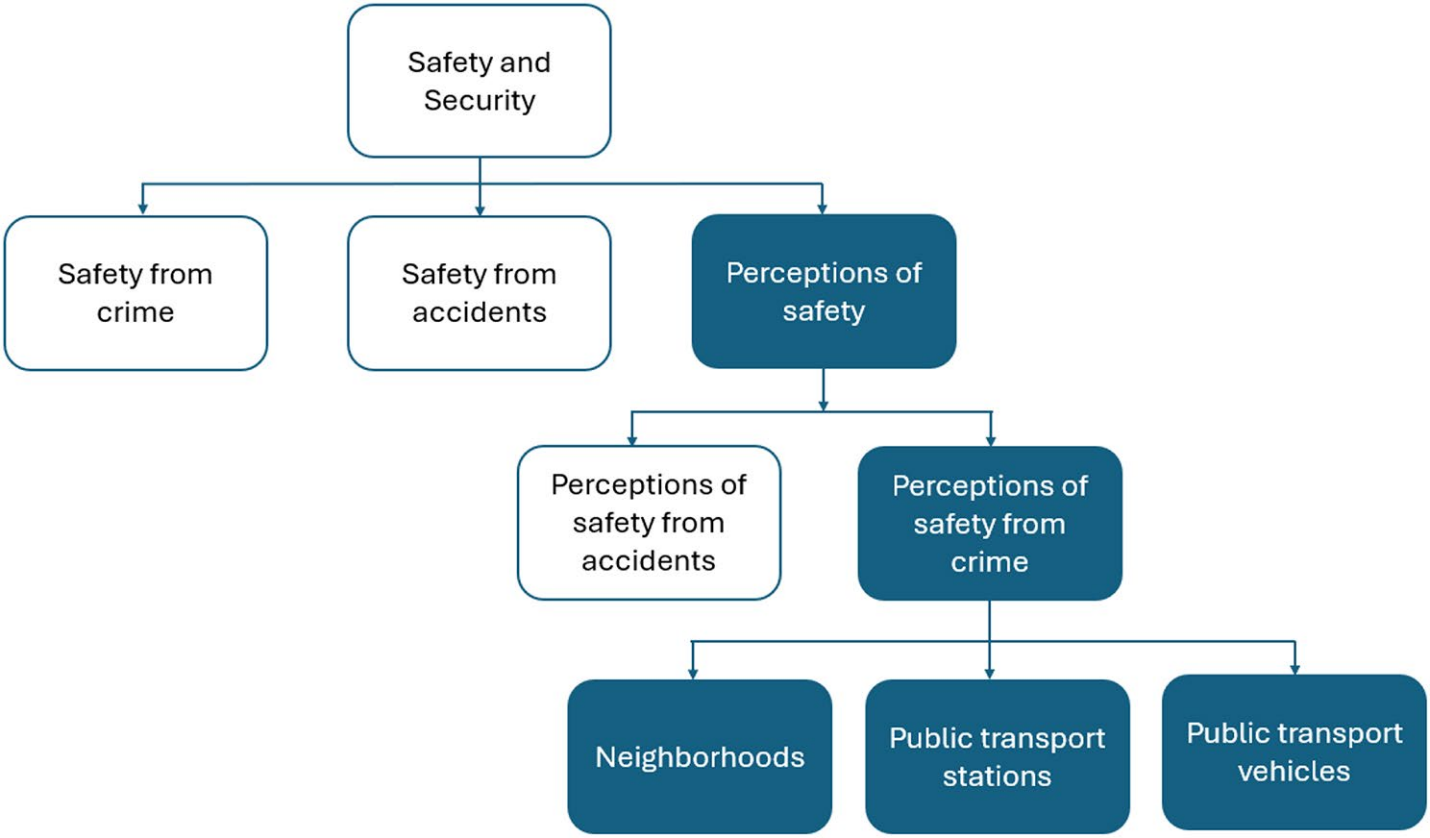
The concepts of safety and security in urban transportation. Source: Masoumi and Fastenmeier (2016)

Mobility is considered as one of the key determinants for productive ageing while keeping the older adults active and engaged within their community [8]. Studies have shown that mobility has a strong association with the health and well-being of older adults [9, 37]. However, inadequacies in the transport infrastructure create a sense of fear for older adults to use different transport modes for mobility. Older adults felt more unsafe than younger age groups while travelling making public transport safety an important concern for their everyday commute [38, 39]. Therefore, safety is regarded as a key issue with respect to transport-based exclusion. Often, perceptions of safety have been discussed with respect to behavioural sciences and criminology [40, 41]. However, for this paper we will focus on perceptions of safety only with respect to transportation. Masoumi and Fastenmeier (2016) adopted the framework from the European Commission (as shown in Fig. 1), which categorized ‘security’ into three themes as follows: (a) safety from crime (b) safety from accidents (c) perceptions of safety [42]. They examined the concept of *perceptions about safety* from crime in urban public transport in Germany. The authors consider perceptions of safety as ‘subjective security’ and refer to it as “the negative perceptions about safety from crime limiting the use of public transport by urban residents” [43]. They identified that neighbourhoods, transport stations, and transport vehicles were areas where users may encounter risks during commute. In addition to the perception of safety from crime, this paper expands the concept of perceived safety by incorporating additional domains of safety such as *perception of safety* with respect to accidents, travel after dark, and the COVID-19 pandemic. We explore various elements in the built environment design (e.g., sidewalks), transport mode (e.g., falls while boarding/alighting, non-violent crime), and transport stations where older adults may encounter risks. Therefore, the current research will help in advancing our understanding about older adults’ perceptions of safety in transportation and how it influenced their mobility.

## Methodology

### Study setting

The study was conducted in Bengaluru (urban district), a rapidly growing metropolitan city in the state of Karnataka, which is in the southern part of India. According to the National Health Systems Resource Centre (NHSRC) report, the population of older adults (above 60 years) in Karnataka is about 11.5% of the total population. The report also indicates that 71% older women and 24% older men were economically fully dependent on others [44]. The total population of Bengaluru is currently estimated to be 14,008,262, and the city hosts the largest population of older adults i.e., 9,11,336 among other districts in Karnataka [45]. Rapid urbanization in the city has resulted in traffic woes and infrastructure related challenges. The city presently has different modes of public transport available such as metro rail, Bengaluru Metropolitan Transport Corporation (BMTC) buses, suburban trains, and intermediate transport.

### Participant profile and selection

**Fig. 2:**
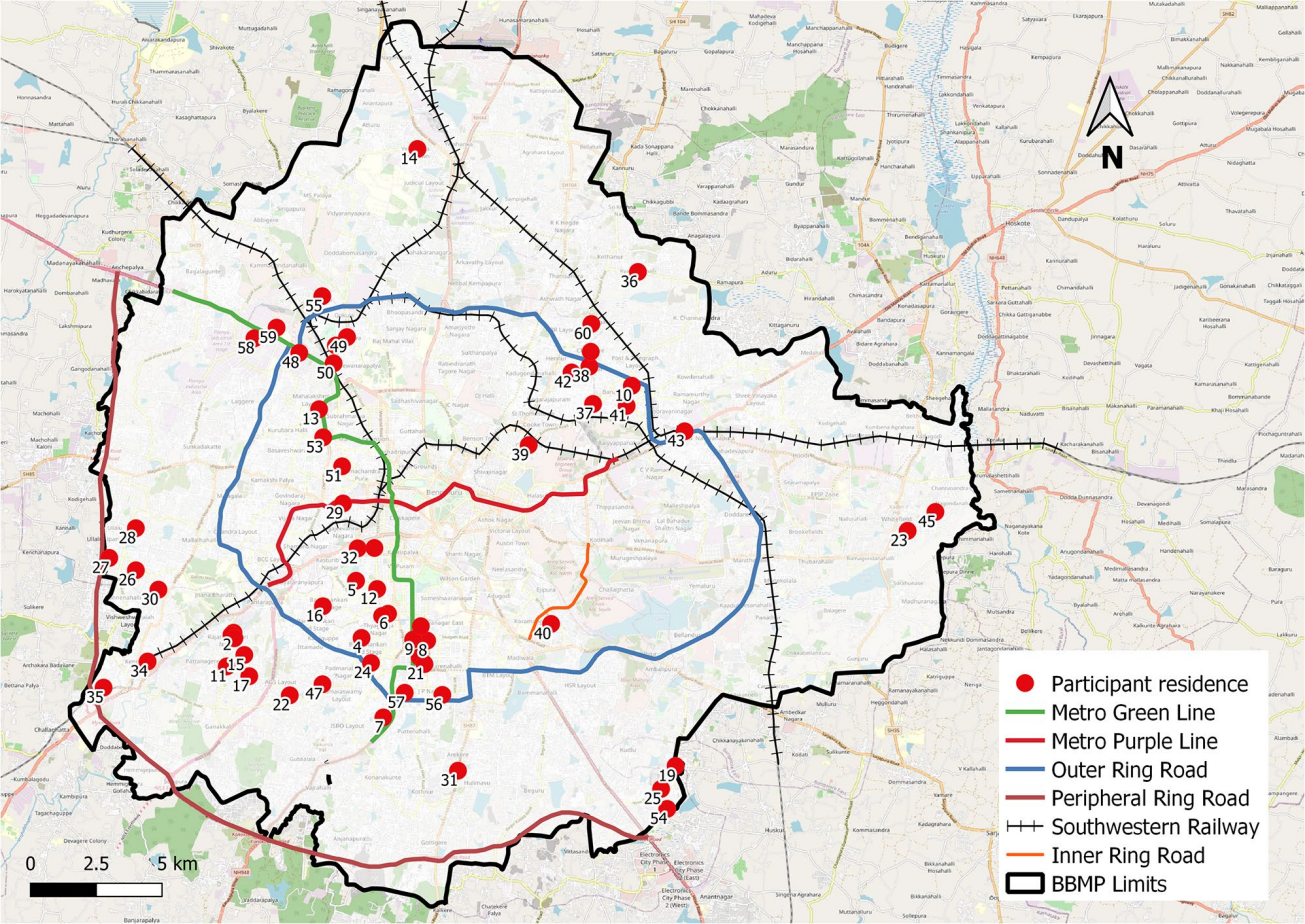
Residential locations of participants in the study

Purposive sampling was used to recruit 60 participants from urban Bengaluru. Inclusion criteria for participants were: (1) older adults (> 50 years) living in Bengaluru. 50 years and above was considered since many working-class people in the pre-retirement stage, begin to make changes with respect to their occupations and mobility behaviour based on their health conditions [46] and (2) not cognitively impaired. To understand a range of experiences participants were recruited from both genders, different socio-economic status, employment status, residential location (see Fig. 2), religion, and living arrangements. The recruitment of participants was not to be representative but to understand the experiences of participants. Of the 60 participants, 34 were males and 26 were females. Many of the participants were retired and those who were working had different occupations. More details on participants’ profiles are given in Table 1.

**Table 1:** Participant profile

	Name	Age	Gender	Marital status	Religion	Occupation	Monthly Income (INR)	Geographical location of residence	Mode of transport used
1	Paramanand	61	Male	Married	Hindu	Retired	30,000–50,000	Bommanahalli	Bus, two-wheeler, cab, metro
2	Basavaraj	63	Male	Married	Hindu	Retired	30,000–50,000	South	Bus, two-wheeler, walk
3	Bharath	65	Male	Married	Hindu	Retired	30,000–50,000	South	Walk, cab/auto
4	Channesh	62	Male	Married	Hindu	Retired	10,000–20,000	RR Nagar	Walk, cab/auto, vehicle driven by someone else
5	Premanand	66	Male	Married	Hindu	Private job post-retirement	40,000–50,000	RR Nagar	Walk, cab/auto, two and four-wheeler
6	Shyam	67	Male	Widower	Hindu	Private job post-retirement	10,000–20,000	East	Walk, bus
7	Rebecca	65	Female	Separated	Christian	Not working	Pocket money (son)	Byataranyapura	Walk, auto, cab
8	Ratna	66	Female	Married	Christian	English teacher in Bible college.	Currently no income (due to COVID-19) Some well-wishers pay them money for basic needs	Byataranyapura	Walk, bus and auto
9	Dorothy	78	Female	Married	Christian	Not working since a year	20,000–40,000	East	Car, walk, auto and bus
10	Amarnath	65	Male	Married	Hindu	Social service post-retirement	60,000–80,000	South	Car, bike, (Very rarely bus/auto/cab)
11	John	65	Male	Married	Christian	Retired	40,000–60,000	Byataranyapura	Bus, cab, auto, walk
12	Sarah (Saroja – paper 2)	63	Female	Widowed	Christian	Not working	Children pay pocket money	East	Bus
13	Rathnakar	58	Male	Married	Hindu	Retired	Retirement income	Mahadevpura	Bus, metro, cab, auto, walk
14	Dayanand	58	Male	Married	Hindu	Consultant	40,000–60,000	South	Metro, bus, two-wheeler, car, cab
15	Anupamma	66	Female	Married	Hindu	Visiting professor/translation	20,000–40,000	East	Bus, local train, car (co-passenger)
16	Sunitha	60	Female	Separated	Hindu	Cleaning (shops)	5000–7000	East	Bus, auto and walk
17	Ashwini	60	Female	Widow	Hindu	Waste segregation worker	< 5000	South	Auto, bus and walk
18	Rajendra	76	Male	Married	Hindu	Security guard	10,000	West	Bus and walk
19	Babu	66	Male	Widower	Hindu	Not working	Nil. Son pays for medicines	West	Bus and walk
20	Chandrika	50	Female	Married	Hindu	Not working. Before Covid broker work sometimes	Nil. Husband pays for expenditure	West	Walk and bus. Cab/auto sometimes
21	Rajshekar	65	Male	Married	Hindu	Not working	20,000–40,000	West	Walk, bus, auto, cab
22	Nazeema	60	Female	Married	Muslim	Home maker	Husband pays for expenses	West	Walk, bus, sometimes auto, cab/metro
23	Nagaraj	67	Male	Married	Hindu	Retired	20,000–40,000	West	Two-wheeler, walk
24	Sandhya	73	Female	Widow	Christian	Retired	5200	South	Bus, cab, auto, walk
25	Suganthi	63	Female	Married	Christian	Working at Private institution (BSI)	20,000–40,000	West	Metro, bus, cab, auto, walk
26	Kalpana	51	Female	Married	Hindu	Not working		South	Car, metro, cab, walk
27	Mohan	80	Male	Married	Hindu	Retired	20,000–40,000	South	Bus, walk, metro/auto sometimes only, car (as passenger)
28	Sreenath	60	Male	Married	Hindu	Not working	less than 5000	Dasarahalli	Walk, bus
29	Nagesh	68	male	Married	Hindu	Private business	5000–10,000	Dasarahalli	Bus, two-wheeler, auto and walk
30	Shalini	55	Female	Married	Christian	Retired	Retired	East	Bus, walk or two-wheeler (pillion rider)
31	Ramesh	68	Male	Married	Hindu	Private business	40,000–50,000	RR Nagar	Two-wheeler and car
32	Raghunath	64	Male	Married	Hindu	Retired	20,000–40,000	RR Nagar	Uses bus
33	Ravi	82	Male	Widowed	Hindu	Retired	40,000–50,000	RR Nagar	Uses bus, ola auto/cab
34	Lakshmi	75	Female	Widowed	Hindu	Home maker	20,000–40,000	South	Usually by walk
35	Anand	68	Male	Married	Hindu	Retired	20,000–40,000	South	Scooter, bike, car
36	Prakash	60	Male	Married	Hindu	Retired	40,000–60,000	South	Scooter, car
37	Vandana	60+	Female	Widowed	Hindu	Domestic helper	6000	South	Walk, bus, auto
38	Venkatesh	60	Male	Married	Hindu	Not working	20,000–30,000	South	Bike, metro
39	Sharada	62	Female	Married	Hindu	Retired	13,000	South	Has a car driver, metro and auto rickshaw
40	Jacob	58	Male	Married	Christian	Retired	20,000–50,000	East	Scooter, Metro, Cabs/Auto
41	Sunandha	65	Female	Widowed	Hindu	Home maker	20,000–40,000	RR Nagar	Hires a driver, auto, walk
42	Pushpa	60	Female	Married	Hindu	Retired	13,000	South	Office van, auto, personal vehicle (co-passenger)
43	Shanta	62	Female	Married	Hindu	Working	50,000–60,000	West	Car (self), driven by husband/driver (car), metro, cabs/auto
44	Sreenivas	65	Male	Married	Hindu	Retired	30,000	Byataranyapura	Bike, cabs
45	Vanita	74	Female	Married	Hindu	Home maker	Dependent on husband	RR Nagar	No private vehicle, uses bus, metro, auto, cabs and mostly walks
46	Lakshmi	62	Female	Widowed	Hindu	Insurance agent	20,000–30,000	East	Cabs and autos
47	Lalitha	62	Female	Married	Hindu	Home maker	50,000+	RR Nagar	Car (driver), car (self), metro and by walk
48	Lokesh	70	Male	Separated	Hindu	Retired	50,000+	South	Car (self), metro, bus (Volvo only), walking
49	Krishnamurthy	60	Male	Married	Hindu	Retired	30,000	South	Car, two-wheeler, metro, bus, by walk
50	Murli	70	Male	Married	Hindu	Retired	40,000+	South	Two-wheeler, metro, cabs, autos, walk
51	Ahmed	78	Male	Married	Muslim	Retired		South	Two-wheeler, cabs.
52	Prema	60	Female	Widowed	Hindu	Domestic maid	7000	RR Nagar	Bus and walk
53	Anitha	78	Female	Separated	Hindu	Chef	7000	Mahadevapura	Bus, auto and walk
54	Aamir	78	Male	Married	Muslim	Retired		South	Auto and travels with son most often.
55	Gopal	60	Male	Married	Hindu	School shoe store	8000–10,000	South	Bus, cycling, walking
56	Nirmala	76	Female	Married	Hindu	Not working	1000 pension	RR Nagar	Bus, walking
57	Narayanan	72	Male	Married	Hindu	Newspaper packing	7000–8000	RR Nagar	Bus, walking
58	Vishwamurthy	76	Male	Married	Hindu	Not working	1000 pension	RR Nagar	Bus, walking, previously cycling
59	Anupamma	70	Female	Widowed	Hindu	Housekeeping work in a factory	3200 salary + 1000 pension	RR Nagar	Walking, auto, bus
60	Hussain	66	Male	Married	Muslim	Auto driver	5000	RR Nagar	Auto, walk

### Data collection

#### Observations

The researcher made observations one month prior to the beginning of Covid-19 pandemic at bus stops in the neighbourhood of Devara Jeevanahalli (also known as DJ Halli). DJ Halli is located in Bengaluru north and closer to the central business district (CBD). The residents in the area belong to socio-economically marginalised population. The bus stops were located at busy junctions, with one of them being the main bus station for the area. The locations were chosen because it was a convenient place for the researcher to wait and make the observations. There was an auto rickshaw stand nearby and vendors selling fruits and vegetables on the pavement. The aim of the observations was to understand the different modes of transportation employed/accessed by older adults, frequencies of buses and intermediate transport, infrastructure of bus stops and metro stations, and difficulties faced by older adults to board and alight from the buses, cross the roads, and walk on the pedestrian pathway. The observations took place between 8:00am and 1:00pm to include both peak and non-peak traffic hours. Notes and photographs were taken during the observations to show the challenges that older adults faced during their daily commute through the city.

#### In-depth interviews

Telephonic in-depth interviews were carried out between June – December 2020 owing to mobility restrictions due to Covid-19 pandemic. There were other studies conducted during the pandemic, which used remote methods such as telephonic interviews to understand the perceptions and experiences of individuals [47–49]. Initial contact with participants was made through the researcher’s known contacts who helped connect with older adults from different income groups (lower-middle, upper-middle, and high income), and with the help of Non-Governmental Organisations (NGOs) such as HelpAge India and Hasirudala which worked among lower-income older adults. This was followed by snowballing. The participants were explained about the research, allowed to ask questions regarding the research, and later asked for their willingness to participate. After obtaining consent, a convenient date and time was fixed for the actual interview.

A semi-structured in-depth interview guide (given in appendix 1) was developed and modified. Trial interviews (*n* = 2) were conducted before beginning the actual interviews. The telephonic (audio call) interviews we recorded using the mobile phone’s in-built voice recorder after obtaining the consent from the participant. Notes were taken by the researcher during the interview. The duration of interviews ranged between 40 and 90 min. Interviews were conducted in English, Kannada, and Tamil languages. We interviewed participants until data saturation.

#### Ethical approval

Ethical approval for the study was obtained from Science-Geosciences Ethics Review Board, Utrecht University (Geo L- 19294) and Institutional Ethics Committee, Institute for Social and Economic Change (DPA/Ethic.Com/2020/343). We used pseudonyms for the names of participants in the text.

### Data analysis

#### Data management

The recorded interviews were transcribed and translated to English. Three interviews for which we faced technical issues with recording, the researcher took notes and elaborated them after the interview. The data were analysed using NVIVO version 12. Coding of interviews was done by the first author in consultation with the second author. A two-stage coding cycle was undertaken [50]. Initially the transcripts were read by DSP to identify the list of deductive and inductive codes. The deductive codes were developed based on the conceptual framework (given in Fig. 1) and inductive codes from the data itself. Key concepts from the conceptual framework are perceptions of safety from accidents, crime, built environment characteristics, public transport, and public transport station. The inductive codes were road infrastructure, pavements, travel after dark, challenges while walking, street lighting, behaviour of transport personnel, and traffic volume. In the second cycle, different codes having similar attributes were grouped under code families to develop themes and sub-themes that were related to safety perceptions around different transportation systems and its influence on the mobility of older adults.

#### Findings

Our research highlighted older adults’ perceptions of safety from accidents, crime, travel after dark, and COVID- 19 at different components of transportation system during their everyday travel. They found it challenging to navigate through the city, which significantly influenced their mobility in the community.

##### Perception of safety from accidents

**Fig. 3:**
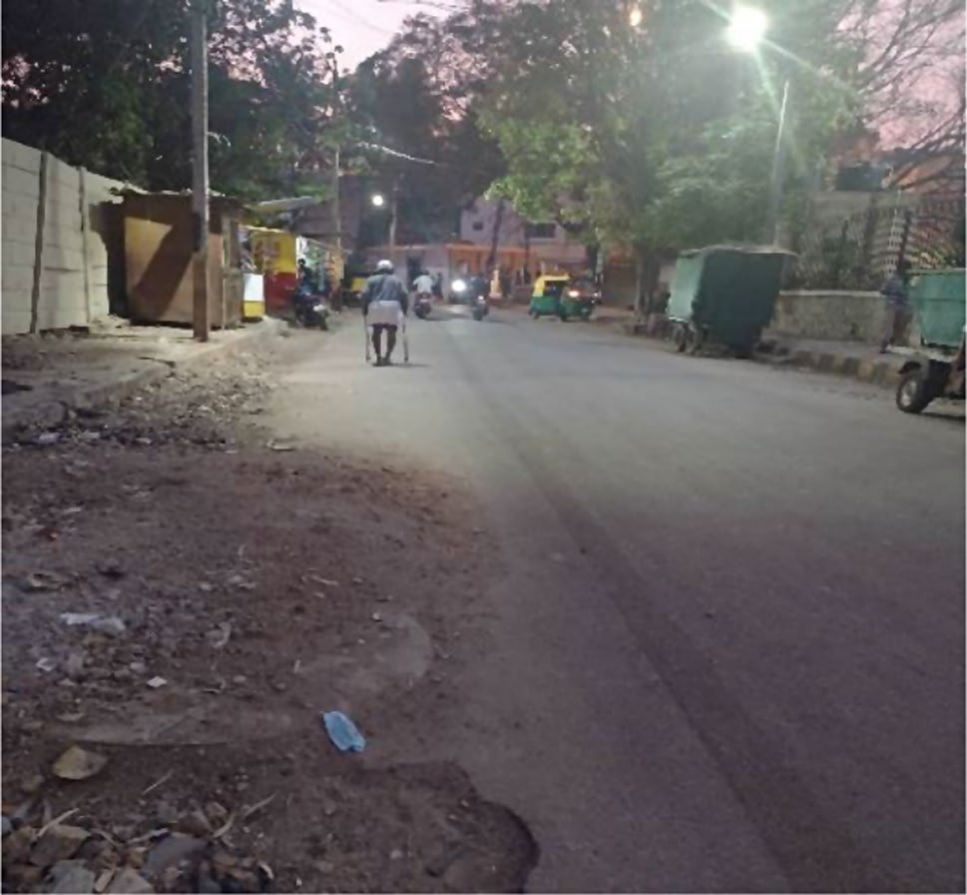
Infrastructure in a low-income neighbourhood

The features in the built environment where older adults commute (e.g., pavements, street design) significantly affected their mobility. Many of our participants mentioned that the roads were of poor design and ill-maintained on the routes travelled by them in the neighbourhood (e.g., grocery store, pharmacy) (see Fig. 3). For example, Bharath, a 65-year-old male, narrated an incident about walking on a dark and poorly lit pavement near his house.

*Unfortunately*,* I went to other footpath. In that house (…) apart from that concrete*,* one more tapered concrete was laid… How I fell down you know ohh… Literally my shoulder and I lost my hopes and all… my house was visible from where I met with the accident… where I fell…*.

This incident resulted in a severe injury to his shoulder, which took several months to heal and still he has difficulty raising his arm completely. Hence, he stopped riding his own vehicle and started to walk, take cab/autorickshaw, or depend on a family member to go outside. After the incident he has stopped going outside the house when it is dark due to poor non-motorised transport (NMT) infrastructure design.

**Fig. 4:**
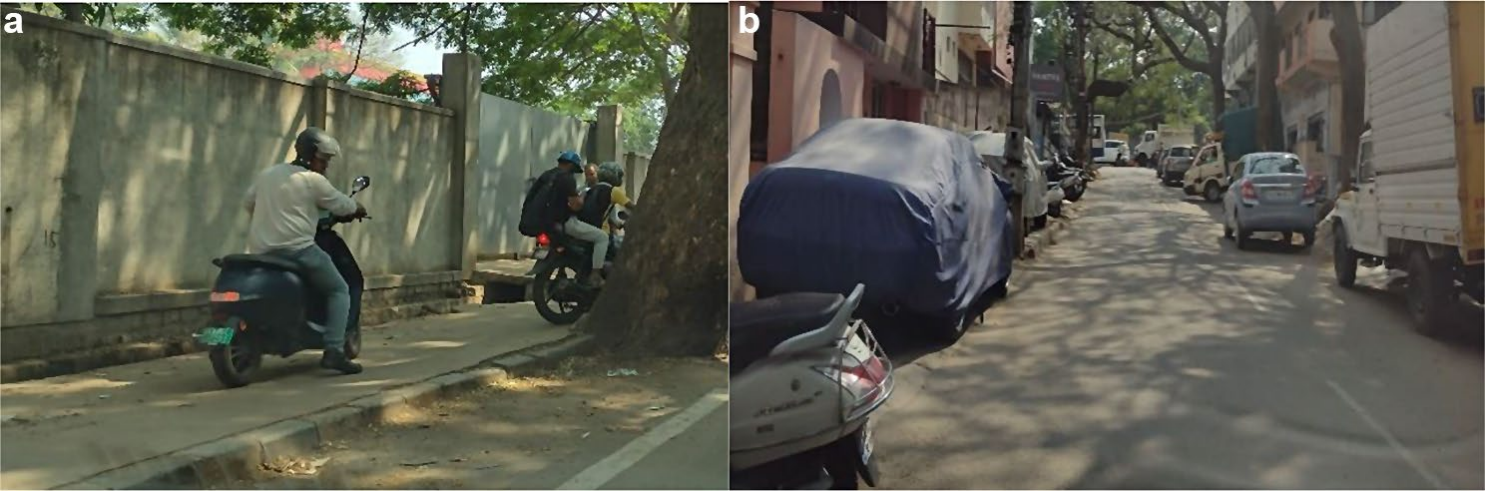
**a** & **b**:Two-wheeler riders on pavement; Cars parked on pavement

In addition, older adults shared experiences of being injured due to accidents while walking on the road, because the pavements were encroached by vendors or parked cars and they were forced to walk on the roads (See Fig. 4a and b). A 60-year-old participant, Prakash shared his experience with motorised two-wheelers riding on pavements as mentioned below

*I would have to tell you this*,* especially in commercial places when there is traffic*,* people straight away take their motorcycles on the footpath. Especially two wheelers. They won’t even be slow; they drive at the same speed as on the road… There have been accidents for older adults because of this… Even some of the car owners will park their cars on the footpath.*

Another common challenge that they faced was that the roads are often dug up for repair works and left incomplete for several months. Such uneven roads resulted in injuries, making mobility challenging for both motorised and NMT users.

*I have tripped on a road. because there was a pack of tar which they had not removed… I had bruises on both my knees. Bruises had turned all black and blue. (Lalitha*,* 62years*,* female)*

Further, perceived lack of safety while crossing the road affected their commute for essential work. Sunita, a 60-year-old woman who works as a housekeeping staff at a store used to walk to commute to work. She found it difficult while crossing the road in the congested traffic without proper pedestrian crossings on the roads in place. Therefore, she had to quit her job. She shared her experience as mentioned below

*Earlier job*,* I used to find it difficult in the morning to cross the road and all that. It caused a lot of problems*,* so I left that job.*

Such experiences limited the mobility of older adults who used walking as a transport mode to carry out errands such as buying essentials, going to work, walking for physical activity, meeting up with friends and family, and participating in activities of social life.

Another important issue that came up pertaining to safety was the erratic road behaviour of bus drivers, which resulted in injuries for older adults due to falls while boarding/ alighting/ mobility in the bus. Pushpa is a 60-year-old female, shared her experience of falling inside the bus while travelling and she suffered fractured ribs.

*I was standing in the bus. The driver applied sudden brake and it was a bad one. I was standing behind*,* but the sudden break made me fall and roll over to the front. Then my ribs were fractured. That was my bad experience.*

Another 65-year-old woman, Rebecca, also shared her experience about having a fall while trying to board the bus.

*Bus is not possible ma [….] Because the drivers are sometimes rash once I fell down also… he started*,* the driver started the bus immediately. I slipped down […] My toe was fractured. My muscle was peeled off in my right hand. I was hurt in my head also. That was the last*,* after that I never went. I am scared.*

**Fig. 5:**
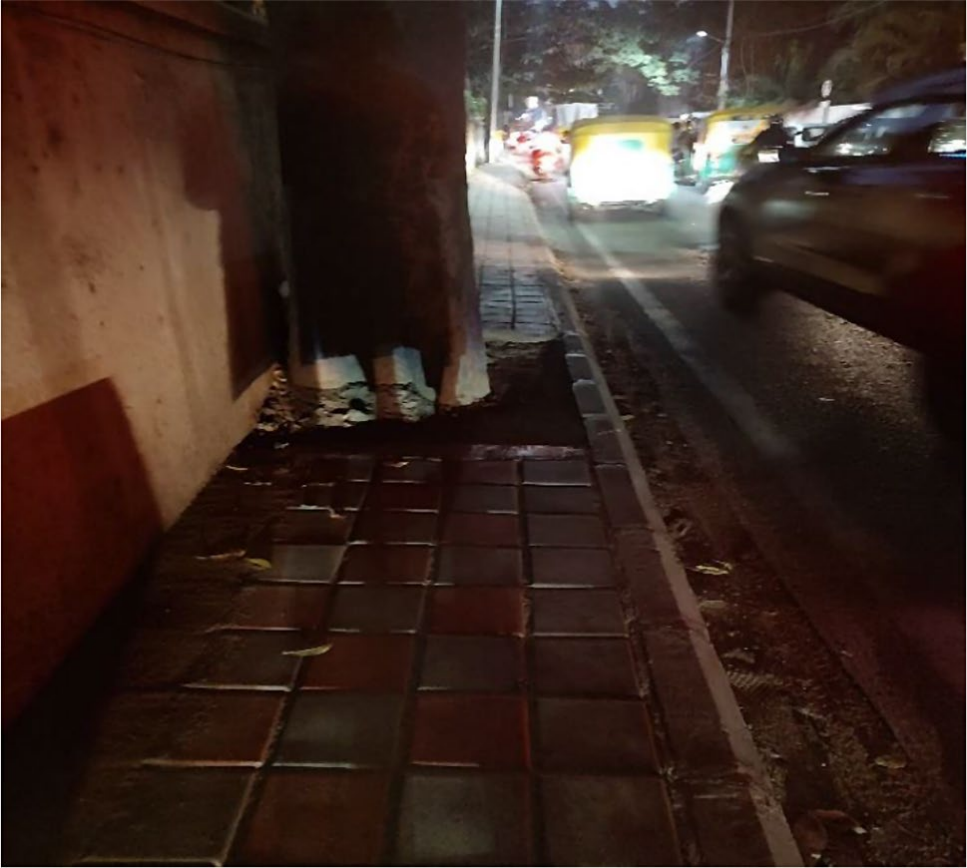
Inadequate lighting in streets

Inadequate lighting on the streets resulted in older adults perceiving a fear of falls and injuries while walking or crossing the roads (see Fig. 5).

*There is no light in that entire road. It is a problem to walk to Masjid on that road. If we have to get up and go in the night (early morning) it will be difficult. (Hussain*,* 66 years*,* male)*

*I don’t go anywhere in the evening. What if I fall somewhere in the dark? That is why I don’t go out. (Vandana*,* 60 years*,* female)*

##### Perception of safety from crime

Mobile thefts and chain snatching in the neighbourhood areas caused a lot of fear among older adults. The state of Karnataka has reported an increase in the number of chain snatching incidents in 2022 compared to previous years [51]. Older women in India usually wear gold chains or gold-plated chains (depending upon their financial status) as a customary practice for their everyday use. They are being taken advantage of being vulnerable and unable to run or call for help. More often older women spoke about incidents of chain snatching and mobile phone thefts that occurred to them in their locality and hence they had the fear of going out for walking (physical activity) where they usually meet up with friends and spend time. For example, Lakshmi, a 62-year-old woman, who lives alone used to go for a walk in the neighbourhood where she meets her friends at the park and spends some time with them. But due to incidents of chain snatching in that locality she and her friends stopped walking regularly. Even if they had to walk, they took precautions such as going in a bigger group and keeping the jewellery at home before stepping out of the house.

*So*,* we all stopped our morning walks after that… If we really want to go*,* then four-five of us will go together. Or we will remove these gold chains and keep them at home before leaving. We have to do all such things.*

*The crime rate has increased quite a lot… Now*,* even during morning walks*,* chains are snatched from old couples*,* old ladies who can’t even run*,* who can’t even shout. (Amarnath*,* 65 years*,* male)*

Incidences of theft were seen within the public transport that created fear and influenced their mobility to carry out daily activities such as work and shopping essentials. Shyam, a 67-year-old participant, who works as a private consultant after retirement, shared his experience of pickpocketing inside the bus.

*See if there is a lot of rush in the bus*,* there will be a lot of pick pocketers*,* they will have their own group and will be standing on the step only. They will push around the people and touch our pockets*,* that is their only job. I have seen this from such a long time now.*

Another participant shared a similar experience inside the bus,

*I was standing*,* and I don’t know how she knocked off my mobile. That day onwards I was very scared to travel on bus. (Sunanda*,* 65 years*,* female)*

Other unsafe and unpleasant encounters that elderly women confronted at the bus stops included the presence of drunk men and improperly/incompletely built bus waiting stations. While waiting at the bus stop Anita, a 78-year-old participant mentioned:

*Aiyo in few places*,* the bus stops won’t even have a shelter to stand under. In the available shelter men would have gotten drunk and be sleeping there.*

Similarly, another older adult highlighted the infrastructure deficit and unpleasant atmosphere around the bus stop. Such unpleasant environments around the bus stops made older adults feel unsafe while waiting for the buses.

*The bus stop is not very good; they have not made it properly. The have left it half done. There is place to sit*,* but you will see only those who are drunk there and fallen… they will sleep there only*,* vomit there or sit there only and drink also. (Sreenath*,* 60 years*,* male)*

While public transportation had reserved seats for women, older women felt unsafe travelling after sunset for the fear of crimes taking place in the neighbourhood or on the transit mode.

*Sometimes when it got late*,* I have felt… men come and sit in ladies’ seat also; it is not good. At such times I don’t feel very secure in the bus. (Shalini*,* 55 years*,* female)*

Incidents such feeling unsafe due to drunk persons in the bus stations and men in the transport modes resulted in older adults avoiding travel after it was dark. They preferred using personal vehicles for mobility or depending on other family members to go outside their homes.

##### Perceptions of safety from COVID-19 infection

COVID-19 had an unprecedented effect on the everyday lives of older adults. The risk of contracting COVID-19 infection if they went outside the house and while using public transport restricted their mobility outside the house. Dorothy a 78-year-old participant mentioned that she is afraid to visit the hospital and tried to stay at home as much as possible.

*Because heart of hearts you are scared if covid people will be there. That kind of fear is there. Try not to go to a hospital or anything… This is something we have never experienced in our life. Night and all we get scared.*

Another 78-year-old participant, Ahmed, felt confined to his house due to the lockdown restrictions and the fear of travelling in a bus during the COVID-19 pandemic. He describes how he felt excluded from doing his regular activities such as buying groceries in the neighbourhood due to the guidelines imposed by the government for older adults.

*Now*,* due to Corona no one is sitting in a bus. Fear…After Corona came*,* it is like there are no other issues. Everything has gone away. No activities… Now the government says above 65 should not come out of the house. There is no space for us outside… The shopkeeper says*,* ‘why did you come [in a raised voice]? you should have sent your kids. You should be quarantine. Don’t come’. He said so*,* when I went last month.*

**Fig. 6:**
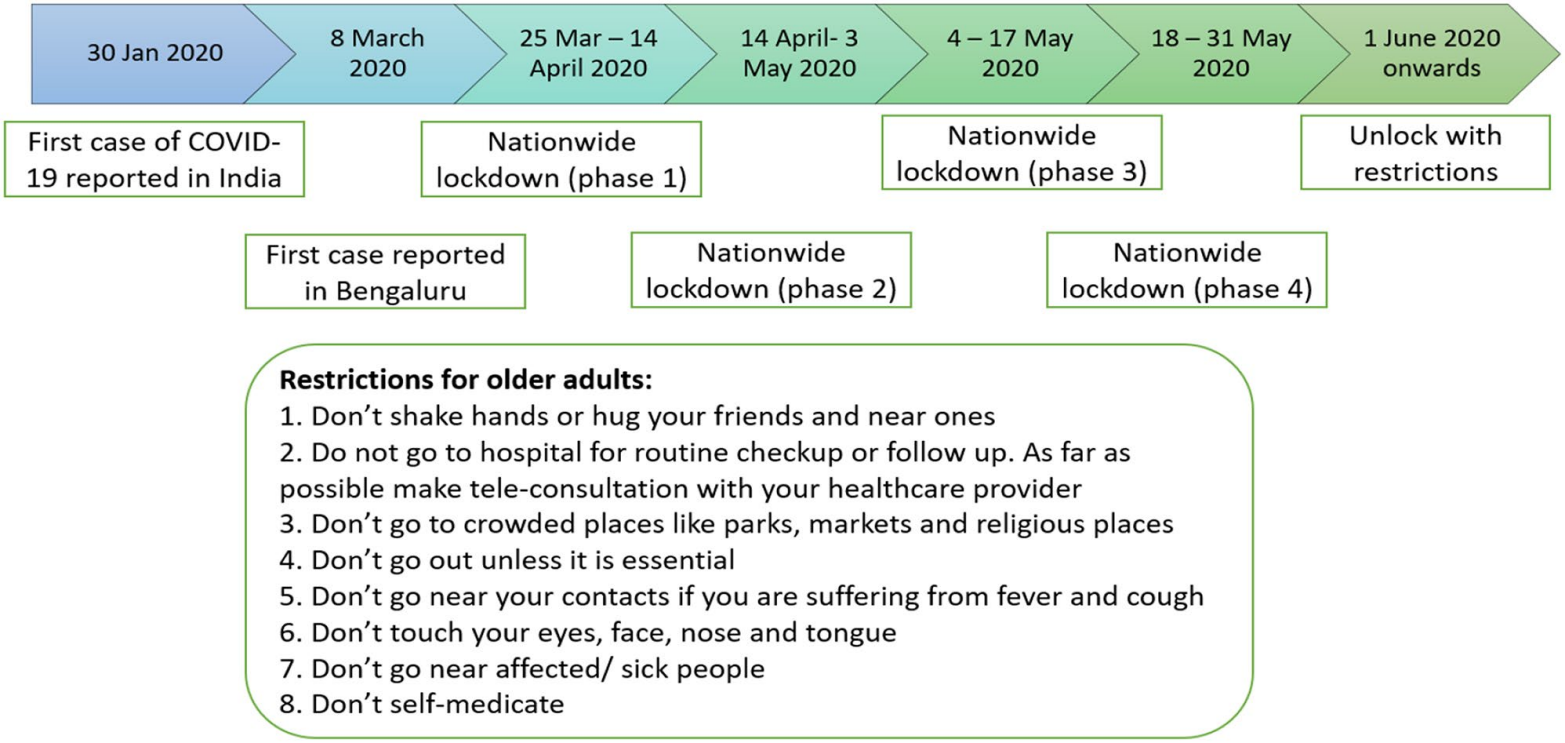
COVID-19 lockdown timeline in Bengaluru [Source: Author]

Figure 6 depicts the COVID-19 lockdown timeline and the restrictions imposed on older adults. The guidelines imposed by the government were sudden giving very little opportunity for older adults to be prepared for it. However, there were participants who felt they had to follow the measures imposed to not get infected. Hence, there was an overall reduction in their mobility outside the house.

## Discussion and implication

**Fig. 7:**
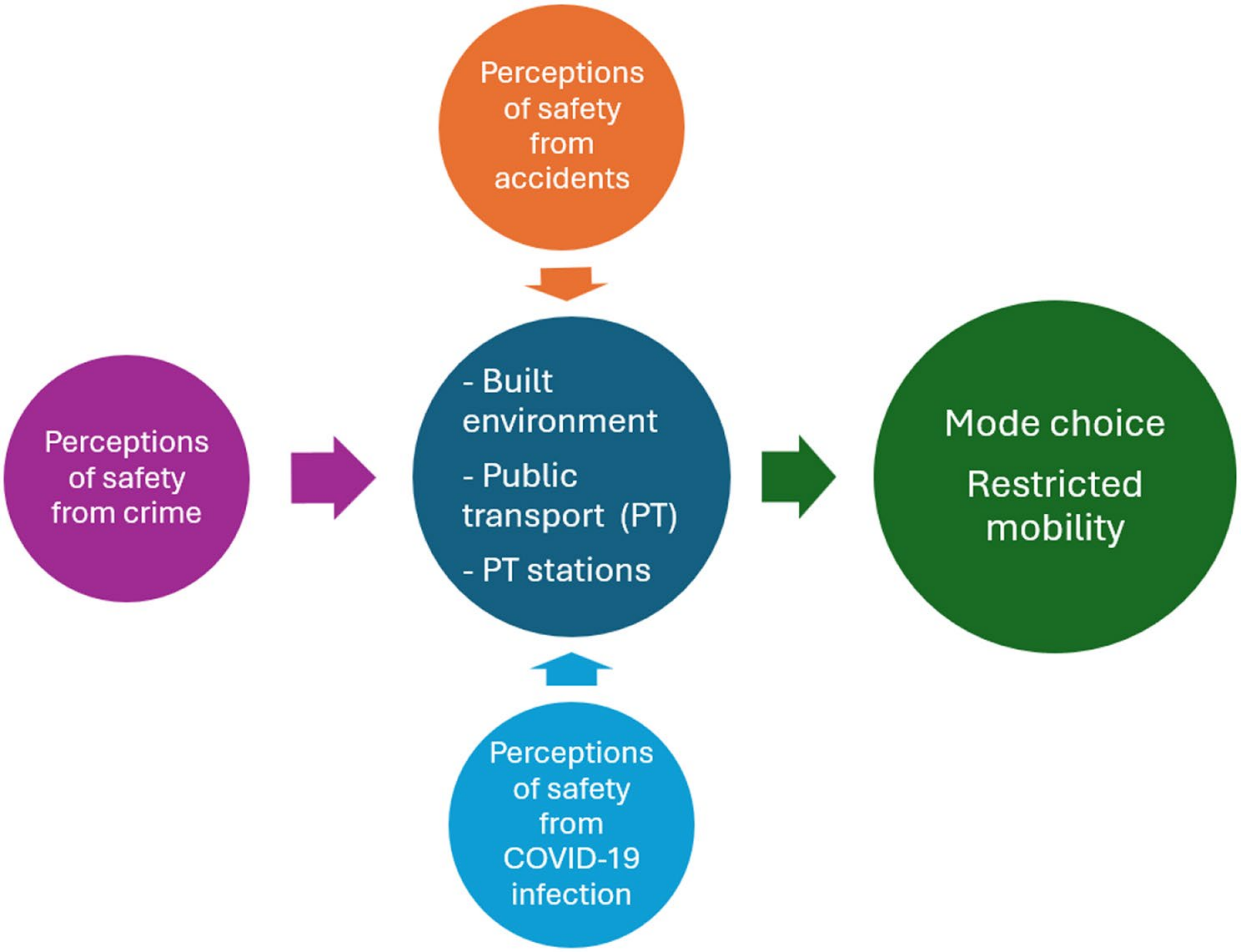
Depiction of perceived safety in the transportation system and mobility of older adults

In our research, we found that older adults were more likely to experience accidents and injuries due to unsafe and inadequately planned transport systems. In this paper, we expand the concept of perceived safety given by previous literature, by incorporating additional domains of safety with respect to transportation. Figure 7 depicts the perceptions of safety from accidents, crime, and COVID-19 pandemic that influenced the mobility of older adults. This framework has been adapted from Masoumi and Fastenmeier (2016) based on the concepts of safety and security in urban transportation [43].

The most common challenge that older adults highlighted in our research was feeling unsafe with respect to built environment features, particularly while walking. Older adults walked for various reasons such as buying groceries, to reach the bus stop or metro station, to visit a doctor’s clinic if it was nearby, and for physical activity/ recreation. Often walking is considered important to maintain physical fitness and good health [52]. However, in our research, walking was an important concern for older adults, which limited their mobility to activities outside their homes. Majority of them emphasised on the fact that though efforts have been made by the local government to improve NMT infrastructure such as construction of pavements and having adequate streetlights, they lack in the maintenance of infrastructure. The roads or pavements are dug up for repair works and left unattended for long periods of time making it unsafe for walking. Challenges due to poor quality walking infrastructure in the built environment such as pavements, street design and lighting, road infrastructure, pedestrian crossings and signals often caused accidents/ physical injuries, fear, and anxiety, among older adults while commuting for daily activities. Providing good quality transport infrastructure such as sidewalks that are clean and well-lit, signals at pedestrian crossings and safety from crime are important factors, which promote walking, reduce transport disadvantage, and improve social inclusion and physical health among older adults [3, 53]. Improved participation in social life and better health outcomes were seen in communities that are transit oriented and have good NMT infrastructure [33, 54]. Available research shows that adhering to the design standards followed universally will help increase participation of older adults in the society. For example, features of the neighbourhood public spaces that promote walking helped older adults in improving social connections and remain socially active in countries such as the United States of America, United Kingdom, South Korea, Japan, Canada, Israel, China, Singapore, Thailand, Germany, and Australia [55]. In addition to these challenges, crimes (mobile phone/ chain snatching) while walking made older adults especially older women feel unsafe even in their own neighbourhood areas. This limited their ability to meet up with friends/family that could result in social isolation affecting their mental and social well-being. Our findings were congruent with research conducted in Hong Kong where crimes such as thefts had a negative impact on walking for older women [56]. Stressful interactions during their commute disrupted their mobility increasing the risk of social isolation and feelings of loneliness [57]. Literature indicated that built environment characteristics such as safety in neighbourhood and covered pavements were factors that enabled older adults’ outdoor mobility [58]. Hence, it is recommended that walking infrastructure should be re-designed according to the requirements of older adults, which will encourage walking among older people. Age-friendly cities framework also emphasises that the outdoor spaces and built environment should be designed to cater to the mobility purposes of the older adults for a making urban mobility more inclusive.

Crimes such as mobile phone theft, pickpocketing, and chain snatching can occur at any time during their commute (e.g., bus stops, on-board public transport etc.). Fear of crime is known to influence the use of public transportation [59, 60]. Our study brought to light that older adults’ felt unsafe while commuting due to thefts (purse/wallet, mobiles, jewellery) inside the transport mode, travelling in the night, and walking in the neighbourhood. As reported by older women, this resulted in a fear of travelling in public transport limiting their mobility to access essential services such as healthcare, leisure activities and work. Our findings highlight the importance of providing services such as security surveillance cameras in public transport modes [61] or security helpline numbers to enhance the safety of older adults when they access public transport to carry out different activities.

Rash driving by bus drivers resulted in falls during boarding/alighting or inside the bus itself, causing physical injuries for older adults. Evidence from high income countries also indicated that older adults got injuries such as minor abrasions, fractures and dislocation of joints while boarding or alighting from the bus, and while standing or moving inside the bus [62]. Often older adults suffer from one or more comorbidities (e.g., hypertension, diabetes). Therefore, it is necessary to ensure that the transport mode they use does not add to their health issues or aggravate already existing ones. Hence, we recommend that the public transport personnel must be sensitised to prioritise the safety of older people when they alight/board and walk inside the transport.

Additionally, increased vehicular traffic inflicted physical injuries to both motorised and NMT users. In places where there were no/ poorly maintained pavements, older people were compelled to walk on the roads alongside speeding vehicles. Older adults expressed feelings of fear while walking or crossing the road due to increased vehicular traffic [63]. These findings point toward reducing the traffic congestion in the city, which could be possible by increasing the number of public transport users.

Since the data was collected during the first wave of the COVID-19 pandemic, the older adults did not want to risk getting infected dreading severe health complications. To remain safe, they opted not to use the public transport, or go outside the house unless it was necessary. They preferred using personal/private transport if there was an emergency. Similar findings were seen across the globe where people were reluctant to use public transport and shifted to personal vehicles during the pandemic [30, 64]. However, for older adults who do not have personal vehicles, measures must be taken to ensure that their mobility needs (e.g., hospital visits, going to work) are met.

The findings from this study should be interpreted with care due to a few limitations. Firstly, the study was carried out on a diverse group of participants residing in various locations of Bengaluru. It should be noted that due to the geographical variation, not all participants may have similar perceptions regarding safety during their everyday travel. Secondly, due to the COVID-19 pandemic, instead of conducting in-person interviews, the researcher had to rely on telephonic interviews. However, this approach presented a challenge for the researcher to observe the participants’ surroundings.

## Conclusion

We identified that safety perceptions around the transport system stem from prior negative experiences during commute and this is largely influenced by age, gender, location, and socioeconomic class. Owing to physical and cognitive decline, and not owning a car/ two-wheeler older adults preferred to walk, use public transport, or depend on others for mobility. Providing easy and safe access to older pedestrians through improved walking infrastructure (for e.g., clean and well-lit sidewalks, signals and markings at pedestrian crossings) will encourage the use of public transport modes, which is a sustainable option to getting around. Furthermore, increased use of public transport will help reducing traffic congestion. Increasing surveillance in the public transport and public transport stations along with security helpline numbers will help older adults use public transportation without fear. Installing comfortable bus shelters, sitting spaces, and lighting inside the shelter can improve the waiting experience for older adults. Among the various aspects within transportation, safety and comfort during commute significantly affects the mobility and well-being of older adults. Therefore, ensuring safety around the components of transport systems such as built environment, within the public transport, and public transport stations is of utmost importance because it shapes the mobility and transport mode choice of older adults and helps promote healthy ageing across the cities.

## Data Availability

Data will be available with the corresponding author on reasonable request.

## Abbreviations

NGOs: Non-governmental organisations
NMT: Non-motorised transport
