# Correction: Perceptions of safety during everyday travel shaping older adults’ mobility in Bengaluru, India

**DOI:** 10.1186/s12889-024-19647-8

**Published:** 2024-08-06

**Authors:** Divya Sussana Patil, Ajay Bailey, Sobin George, Lena Ashok, Dick Ettema

**Affiliations:** 1 Transdisciplinary Centre for Qualitative Methods, Department of Data Science, Prasanna School of Public Health, Manipal Academy of Higher Education, Manipal, Udupi, Karnataka 576104 India; 2 Department of Human Geography and Spatial Planning, Faculty of Geosciences, Utrecht University, Utrecht, The Netherlands; 3 Centre for Study of Social Change and Development, Institute for Social and Economic Change, Bengaluru, India; 4 Department of Social and Health Innovation, Prasanna School of Public Health, Manipal Academy of Higher Education, Manipal, Karnataka 576104 India

**Correction to: BMC Public Health (2024) 24:1940**

10.1186/s12889-024-19455-0

The original publication of this article contained an anonymized approval section. The incorrect and correct information is listed in this correction article, the original article has been updated.

Incorrect:

Ethical approval for the study was obtained from XXX (Geo L- 19***) and XYZ (DPA/Ethic.Com/2020/***). We used pseudonyms for the names of participants in the text.

Correct:

Ethical approval for the study was obtained from Science-Geosciences Ethics Review Board, Utrecht University (Geo L- 19294) and Institutional Ethics Committee, Institute for Social and Economic Change (DPA/Ethic.Com/2020/343). We used pseudonyms for the names of participants in the text.

